# Surgical Application of Human Amniotic Membrane and Amnion-Chorion Membrane in the Oral Cavity and Efficacy Evaluation: Corollary With Ophthalmological and Wound Healing Experiences

**DOI:** 10.3389/fbioe.2021.685128

**Published:** 2021-06-10

**Authors:** Stéphane Odet, Aurélien Louvrier, Christophe Meyer, Francisco J. Nicolas, Nicola Hofman, Brice Chatelain, Cédric Mauprivez, Sébastien Laurence, Halima Kerdjoudj, Narcisse Zwetyenga, Jean-Christophe Fricain, Xavier Lafarge, Fabienne Pouthier, Philippe Marchetti, Anne-Sophie Gauthier, Mathilde Fenelon, Florelle Gindraux

**Affiliations:** ^1^Service de Chirurgie Maxillo-Faciale, Stomatologie et Odontologie Hospitalière, CHU Besançon, Besançon, France; ^2^Université Bourgogne Franche-Comté, INSERM, EFS BFC, UMR 1098, RIGHT Interactions Greffon-Hôte-Tumeur/Ingénierie Cellulaire et Génique, Besançon, France; ^3^Laboratoire de Nanomédecine, Imagerie, Thérapeutique EA 4662, Université Bourgogne Franche-Comté, Besançon, France; ^4^Regeneration, Molecular Oncology and TGFβ, IMIB-Arrixaca, Murcia, Spain; ^5^Deutsche Gesellschaft für Gewebetransplantation (DGFG), Hannover, Germany; ^6^Pôle Médecine Bucco-dentaire, Hôpital Maison Blanche, CHU Reims, Reims, France; ^7^Université de Reims Champagne Ardenne, Biomatériaux et Inflammation en Site Osseux, Pôle Santé, URCA, BIOS EA 4691, UFR d’Odontologie, Reims, France; ^8^Université de Reims Champagne Ardenne, Biomatériaux et Inflammation en Site Osseux, Pôle Santé, URCA, HERVI EA3801, UFR de Médecine, Reims, France; ^9^Chirurgie Maxillo-Faciale – Stomatologie – Chirurgie Plastique Réparatrice et Esthétique - Chirurgie de la main, CHU de Dijon, Dijon, France; ^10^Université Bourgogne Franche-Comté, Besançon, France; ^11^Univ. Bordeaux, INSERM, BIOTIS, U1026, Bordeaux, France; ^12^CHU Bordeaux, Service de chirurgie orale, Bordeaux, France; ^13^Établissement Français du Sang Nouvelle-Aquitaine, Bordeaux, France/INSERM U1035, Université de Bordeaux, Biothérapie des Maladies Génétiques Inflammatoires et Cancers (BMGIC), Bordeaux, France; ^14^Établissement Français du Sang Bourgogne Franche-Comté, Besançon, France; ^15^CNRS, INSERM, UMR-9020-UMR-S 1277 Canther, Banque de Tissus CHU Lille, Lille, France; ^16^Service d’ophtalmologie, CHU Besançon, Besançon, France; ^17^Service de Chirurgie Orthopédique, Traumatologique et Plastique, CHU Besançon, Besançon, France

**Keywords:** amniotic membrane, oral and maxillo-facial surgery, oral mucosa, ophthalmology, wound healing

## Abstract

Due to its intrinsic properties, there has been growing interest in human amniotic membrane (hAM) in recent years particularly for the treatment of ocular surface disorders and for wound healing. Herein, we investigate the potential use of hAM and amnion-chorion membrane (ACM) in oral surgery. Based on our analysis of the literature, it appears that their applications are very poorly defined. There are two options: implantation or use as a cover material graft. The oral cavity is submitted to various mechanical and biological stimulations that impair membrane stability and maintenance. Thus, some devices have been combined with the graft to secure its positioning and protect it in this location. This current opinion paper addresses in detail suitable procedures for hAM and ACM utilization in soft and hard tissue reconstruction in the oral cavity. We address their implantation and/or use as a covering, storage format, application side, size and number, multilayer use or folding, suture or use of additional protective covers, re-application and resorption/fate. We gathered evidence on pre- and post-surgical care and evaluation tools. Finally, we integrated ophthalmological and wound healing practices into the collected information. This review aims to help practitioners and researchers better understand the application of hAM and ACM in the oral cavity, a place less easily accessible than ocular or cutaneous surfaces. Additionally, it could be a useful reference in the generation of new ideas for the development of innovative protective covering, suturing or handling devices in this specific indication. Finally, this overview could be considered as a position paper to guide investigators to fulfill all the identified criteria in the future.

## Introduction

The human amniotic membrane (hAM), or amnion, is the innermost layer of fetal membranes. It is composed of a single layer of epithelial cells, a basement membrane, and an avascular stroma, underlayered by the chorion. The thickness of human term amnion varies among individuals and depends on the location of the sample (70–180 μm thick) (Chen et al., 2012; Gremare et al., 2019). Both amnion and chorion contain mesenchymal stromal cells (MSCs) (Parolini et al., 2008) and variable quantity of growth factors depending on the fetal membranes (McQuilling et al., 2017) and/or the preservation methods (Russo et al., 2011; McQuilling et al., 2017). Basic preservation methods for hAM are cryopreservation, lyophilization and storage in a dry form (Jirsova and Jones, 2017), with questionable cell survival after the cryopreservation process despite the addition of cryoprotective agents (Laurent et al., 2014).

The beneficial effects of hAM have been widely described in the literature. It is immunotolerant, biocompatible and has suitable mechanical properties (permeability, stability, elasticity, flexibility, and resorbability) (Chen et al., 2012). Additionally it possesses anti-fibrotic (Ricci et al., 2013), anti-scaring (Mamede et al., 2012), anti-microbial (Chen et al., 2019) anti-inflammatory (Bailo et al., 2004; Wolbank et al., 2007) and analgesic properties (Rama et al., 2001; Dua et al., 2004; Gajiwala and Gajiwala, 2004). It modulates angiogenesis, having both pro- and anti-angiogenic properties (Mamede et al., 2012; Gholipourmalekabadi et al., 2019) and induces epithelialization and wound healing (Mamede et al., 2012; Gholipourmalekabadi et al., 2019). Finally, it has a low immunogenicity (Kubo et al., 2001), which makes it suitable as an allograft.

To date, ophthalmology is one of the most popular applications of hAM in routine use. The tissue is used as a graft, spread onto the ocular surface to treat epithelial defects or ulcers, or as a bandage to cover the ocular surface to promote healing (Dua et al., 2004; Gomes et al., 2005; Baradaran-Rafii et al., 2007). Several reports have described its use as a covering for the management of wound healing (treatment of chronic ulcers, full and partial thickness burns, skin graft donor sites, over grafting and wounds) (Singh et al., 2004; Mermet et al., 2007; Insausti et al., 2010; Valiente et al., 2018). In both indications, hAM application was facilitated by the access to the tissues being treated (eyes and skin) and by an appropriate description of hAM grafting or covering in the literature.

Since the mid-1990s, there has been a growing interest in using hAM for oral surgery to accelerate tissue regeneration. Chorion and amnion-chorion membrane (ACM) products are also very popular in this indication because they provide not only better handling and thickness, but also provide intrinsic growth factors (Koob et al., 2015; McQuilling et al., 2017). Two recent systematic reviews examined hAM, chorion and ACM benefits for wound healing in various areas of oral reconstruction (Fenelon et al., 2018a; Gulameabasse et al., 2020).

The antimicrobial characteristics of hAM - its ability to manage bacterial infection and biofilm growth and its ability to promote epithelialization - are fundamental properties that benefit to these three tissue sites. The oral cavity and eye share the common property of being in a moist environment with constant movements; severe aqueous tear deficiency, or dry eye, is one major limiting factor for successful hAM transplantation (Shimazaki et al., 2000; Santos et al., 2005). On the other hand, both oral and dermal mucosa exhibit similar macroscopic epithelialization during the wound healing process, which proceeds much faster with a lower inflammatory response and relatively little to no scar formation for oral wounds (Turabelidze et al., 2014).

We have accumulated considerable evidences on hAM use for bone regeneration from experimental studies (Gindraux and Obert, 2010; Obert et al., 2012; Gindraux et al., 2013; Laurent et al., 2014; Gindraux et al., 2017; Laurent R. et al., 2017; Fenelon et al., 2018b; Gindraux et al., 2018; Fenelon et al., 2019; Gualdi et al., 2019; Fenelon et al., 2020; Etchebarne et al., 2021; Fénelon et al., 2021). The diversity of the conditions and methods of membrane application make it difficult to find relevant information in the literature on surgery and tools to judge its efficacy.

The aim of this review is to help practitioners better understand hAM and ACM applications in the oral cavity. The healing effectiveness of fetal membranes has been widely addressed (Fenelon et al., 2018a; Gulameabasse et al., 2020). We focused on clinical hAM/ACM implantation or use as a covering, storage format, application side, size and number, multilayered use or folding, suturing or use of additional protective covers, re-application and resorption/rate. At the same time, we gathered evidence on pre- and post-surgical care and evaluation tools. Finally, we compared our collected information to ophthalmological and wound healing practices.

In an original way, this overview could generate new ideas for the development of innovative hAM/ACM protective covering, suturing or handling devices in the chosen indication. Most important, it could be used as position paper to guide investigators to fulfill all the identified criteria in the future.

## HAM and ACM Application in Oral Surgery

### Application Method: Implantation or Covering Options

In the oral cavity, there are no homogeneous naming practices or consensus about hAM/ACM application. Two procedures have been described: we termed the first “implanted membrane” when the graft was buried beneath the gingiva and the second, “covering graft material” when it was used to cover a mucosal defect and left exposed in the mouth. In the first case, hAM/ACM have guided tissue or bone regeneration (GTR/GBR) membrane functions for periodontal surgery, wound management after implant surgery, bisphosphonate-related osteonecrosis of the jaw (BRONJ) and Schneider membrane perforations repair (Table 1). In the second, hAM/ACM has been incorporated in mandibular vestibuloplasty or oronasal fistulae management (Table 2).

**TABLE 1:** hAM and ACM used as an implanted membrane.

**hAM or ACM**	**Indication**	**No. of patients**	**Graft storage format**	**Graft size (mm)/Side**	**Multilayered use/folding**	**Pre- and post-surgical care**	**Fate/Resorption**	**References and year**
hAM	Gingival recession	1	Lyophilized or dehydrated	Adapted to size of defect	No	Mouth rinses with Chlorhexidine	ND	Shetty et al., 2014
	Gingival recession	3	Dehydrated and sterilized	Adapted to size of defect	No	Antibiotics No tooth brushing for 3 weeks Mouth rinses with Chlorhexidine for 3 weeks	ND	Sharma and Yadav, 2015
	Gingival recession	15	Lyophilized and irradiated (rq: dehydrated written in title)	10 × 10 mm (?) (rq: written 1 × 1 mm in the article)	No	Mouth rinses with Chlorhexidine just before surgery and continued after surgery	Improvement in width of keratinized gingiva at 3 months and 6 months	Jain et al., 2017
	Gingival recession	10	Lyophilized and irradiated	Adapted to size of gingival recession	No	Pre-surgical mouth rinses with Chlorhexidine Mouth rinses for 4 weeks No tooth brushing for 4 weeks Suture removal at day 10	ND	Rehan et al., 2018
	Bone defect in the furcation	5	Lyophilized and gamma-irradiated	Adapted to size of defect	No	Antibiotics for 1 week Mouth rinses with Chlorhexidine twice a day for 4 weeks Suture removal at day 7	ND	Kothiwale et al., 2009
	Bone defect in the furcation	1	Lyophilized and irradiated	Adapted to size of bone defect	No	Antibiotics Mouth rinses with Chlorhexidine for 1 week	ND	Kumar et al., 2017
	Bone defect in the furcation	15	Dehydrated or lyophilized (?) according to figure 4 in the article	Adapted to size of bone defect	No	Antibiotics for 5 days Mouth rinses with Chlorhexidine 2 times a day for 2 weeks Tooth brushing with a soft toothbrush Suture removal at day 7	ND	Kaur and Bathla, 2018
	Intrabony defect in interproximal areas	30	Lyophilized and irradiated	30 × 30 mm	No	Antibiotics for 1 week Suture removal at 1 week	ND	Kumar et al., 2015
	Intrabony defects in interproximal areas	10	Lyophilized and irradiated	Adapted to size of defect	Bio-Oss bone allograft covered with a double layer of hAM, with excess folded	Antibiotics for 1 week Mouth rinses with Chlorhexidine for 4 weeks No tooth brushing for 2 weeks, then only using an extra-soft toothbrush Suture removal at 2 weeks	ND	Kiany and Moloudi, 2015
	Surgical wound after implant surgery	15	Cryopreserved	Adapted to size of wound Mesenchymal side in contact with wound	No	Antibiotics for 1 week Mouth rinses with Chlorhexidine for 2 weeks	At 3 and 6 days: Epithelialization obtained faster on the hAM side than on the standard procedure side	Velez et al., 2010
	BRONJ	2	Cryopreserved	30 × 30 mm	No	ND	ND	Ragazzo et al., 2018
ACM	Alveolar ridge preservation	20	Deepithelialized and dehydrated (irradiated?)	Adapted to size of defect	No	No antibiotics	Epithelialization in 2 weeks approximately	Hassan et al., 2017
	Alveolar ridge preservation	2	Deepithelialized and dehydrated (irradiated?)	8 × 8 mm	No	Pre-operative care: mouth rinses with Chlorhexidine for 3 days Antibiotics for 5 days Tooth brushing with tap water	After 1 month: mature epithelium	Cullum and Lucas, 2019
	Alveolar ridge preservation	21	Dehydrated	Adapted to size of defect	No	Prophylactic antibiotics and mouth rinses with Chlorhexidine before surgery Antibiotics for 10 days Mouth rinses with hydrogen peroxide for 2 weeks	Epithelialization in 2 weeks	Faraj et al., 2020
	Peri-implantation wound management	15	Deepithelialized, dehydrated and irradiated	Adapted to size of gingiva around implant	No	Avoid injuries Mouth rinses (not with Chlorhexidine)	After 2 weeks: the environment around the implant is covered with keratinized gingiva	De Angelis et al., 2019
	Intrabony defect in interproximal area	1	Deepithelialized and dehydrated	25 × 15 mm ACM was cut in half: one half was placed on the root surface. FDBA was covered by the other half	Double layer of ACM around FDBA, the first layer was folded on the grafted bone before applying the second layer	Antibiotics for 1 week Mouth rinses with Chlorhexidine for 2 weeks No tooth brushing for 2 weeks at the site	ND	Hamada et al., 2020
	Schneider membrane perforation repair	9	Deepithelialized and dehydrated (irradiated?)	Adapted to size of perforation, 3 mm wider	2 layers with combination of particulate bone allograft and bone xenograft in between	Antibiotics for 10 days Nasal decongestants: Oxymetazoline nasal spray for 3 days, Pseudoephedrine for 1 week	ND	Holtzclaw, 2015

*Number of applications = 1. ND: not done; ? = information assumed by the authors according to article content; FDBA: freeze-dried bone allograft.*

**TABLE 2:** hAM used as a covering graft material.

**Indication**	**No. of patients**	**Graft storage format**	**Graft size (mm)/Side**	**Multilayered use/Folding**	**Post-surgical care/Additional covering to protect hAM**	**Fate/Resorption**	**References and year**
Mucosal defect after excision of oral submucous fibrosis	25	Fresh	Adapted to the size of the defect	No	Nasogastric feeding for 1 week	Epithelialization (with no indication about the time)	Lai et al., 1995
Mucosal defect after excision of cancerous/precancerous lesions	10	Dried and irradiated	Various sizes: from 21 × 18 mm to 60 × 35 mm mesenchymal side facing the wound	No	hAM placed directly on the wound, stabilized using a pressure dressing of antibiotic ointment gauze removed at day 6 Oral feeding	At day 6: hAM nearly invisible Full epithelialization obtained in a maximum of 6 weeks	Arai et al., 2012
Mucosal defect after excision of cancerous lesions	50	Cryopreserved	100 × 100 mm, then adapted to size of defect	No	ND	At 3 weeks: good granulation tissue formation At 1 month: good surface epithelialization	Khademi et al., 2013
Mucosal defect after excision of cancerous/precancerous lesions	34	Cryopreserved	40 × 40 mm	No	Nasogastric feeding for 1 week	At 3 months: good epithelialization in 100% of patients	Kar et al., 2014
Mucosal defect after excision of benign or precancerous lesions	5	Autologous oral mucosal epithelial cell cultured on de-epithelialized cryopreserved hAM	Adapted to size of defect	No	Suture removal at 1 week	At 1 month: full epithelialization	Amemiya et al., 2015
Mandibular vestibuloplasty	20	Lyophilized and irradiated	Adapted to size of defect identical mesenchymal side facing the wound	No	No stent Tight compression dressing over the lower lip	Day 10: hAM is not differentiated from the surrounding tissues; epithelialization continuation in the graft After 2 weeks: complete resorption of hAM After 3 weeks: graft zone covered by oral mucosa After 4 weeks: complete recovery of the graft	Guler et al., 1997
Mandibular vestibuloplasty	7	Fresh	60 × 100 mm^2^, then adapted to size of defect mesenchymal side facing the periosteum	No	Protection with a splint covered with a tetracycline topical gel and fixed with sutures, removed after 1 week	After 2 weeks: persistence of small segments of hAM After 3 weeks: complete resorption of the hAM, grafted zone still identifiable After 3 months: no difference between the grafted zone and the surrounding mucosa	Samandari et al., 2004
Mandibular vestibuloplasty	10	Preserved in glycerol	60 × 100 mm^3^, then adapted to size of defect mesenchymal side facing the periosteum	No	Use of a suction catheter stent fixed to surrounding mucosa, removed after week 1	After 3 weeks: complete resorption of the hAM, grafted zone still identifiable After 3 months: no difference between grafted zone and surrounding mucosa	Sharma et al., 2011
Mandibular vestibuloplasty	10	Preserved in glycerol	60 × 100 mm, then adapted to size of defect	No	Protection with a splint secured with bone screws, removed at day 7, with cleaning of the surgical site	ND	Kothari et al., 2011
Mandibular vestibuloplasty	2	Dried and irradiated	40 × 20 mm, then adapted to size of defect	No	Surgical splint fixed over the hAM (with mini-screws) and removed at 1 week Oral feeding started the day after surgery	At 1 week: hAM nearly invisible Full epithelialization obtained in a maximum of 6 weeks, with sufficient keratinized gingiva	Tsuno et al., 2014
Oronasal fistulae	4	Cryopreserved	50 × 50 mm, then adapted to size of defect	5 layers	Protection with a palatal plate	ND	Rohleder et al., 2013

*Number of applications = 1. ND: not done.*

When used as *an **implanted membrane***, the main application remains periodontal surgery. Four studies looked at its use as root coverage for gingival recessions, among which three had a coronally advanced flap (Shetty et al., 2014; Jain et al., 2017; Rehan et al., 2018). In these cases, the hAM was applied on the root surface and the flap was sutured over it, taking care not to move or fold it. Sharma and Yadav (2015) raised a partial thickness flap with a double papilla flap technique, with the hAM being applied against the root surface underneath the flap and the suturing performed above it. Furthermore, hAM was used in bone furcation defects in three studies. Some authors first raised a full thickness flap to make a bone graft in the furcation defect and then covered the bone graft using hAM (Kothiwale et al., 2009; Kumar et al., 2017). In these studies, hAM was adapted to the size of the defect at the surgical site; stability was obtained with a drop of saline solution to prevent the hAM from shriveling. Kaur and Bathla (2018) did the same procedure, but with a plasma-rich in fibrin (PRF) membrane instead of a bone graft in the furcation and covered it with hAM. In three studies, hAM was used in vertical interproximal bone loss. In one study, a full-thickness flap was raised and the hydroxyapatite filled defect was covered with hAM; the flap was sutured above the amnion (Kumar et al., 2015). Similarly, in another study, Bio-Oss bone xenograft was covered with a double layer of hAM and the excess folded; the suture was placed above the grafted area (Kiany and Moloudi, 2015). In a different approach, a full-thickness flap was raised and ACM potentialized with a drop of saline combined with freeze-dried bone allograft (FDBA) were applied on the root surface (Hamada et al., 2020). The most coronal part of the ACM, extending from approximately 3 mm over the cement-enamel junction, was then folded over the FDBA. Another layer of ACM was then applied over the ACM/FDBA pair before flap suturing. Finally, three studies tested ACM in alveolar ridge preservation. After extracting the teeth and raising full-thickness flaps, the sockets were filled with demineralized FDBA (DFDBA) covered with ACM, which was tucked in the vestibular and lingual parts of the flap; the gingiva was sutured above it (Faraj et al., 2020). In one study, ACM was secured by sutures; primary closure was not expected, leaving the ACM deliberately exposed (Hassan et al., 2017). In another one, a mix of hydroxyapatite and particulate mineralized bone xenograft covered with ACM was grafted and the gingiva was sutured over it in an inverse “Figure of 8”, providing excellent stability (Cullum and Lucas, 2019).

Two studies investigated hAM or ACM use in wound management after implant surgery. hAM was applied after dental implant placement and the gingiva was sutured above it (Velez et al., 2010) or a full-thickness flap around the implants was raised and ACM was applied around the implant under the periosteum (De Angelis et al., 2019). The membrane was secured using polytetrafluoroethylene (PTFE) sutures and left exposed during the healing phase.

In an original way, hAM was investigated for the management of BRONJ (Ragazzo et al., 2018). After a mucoperiosteal incision with mesial and distal drainage, as well as excision of the necrotic and infected tissues, hAM was positioned on the bone, with the gingiva sutured tightly above it.

Finally, ACM was tested to repair Schneider membrane defects after perforations due to manual hand instrumentation in maxillary sinus augmentation procedures with a lateral approach window (Holtzclaw, 2015). Using the same approach as the surgery that created the perforation, a first layer of dried ACM was applied in the sinus against the perforation and extended by 3 mm from the defect. Once hydrated with blood, the ACM self-adhered well, allowing the investigators to leave it un-sutured. Particulate bone allograft was combined with bone xenograft and covered with another layer of ACM before closing by flap suture.

When used *as **covering graft** material*, five studies have investigated hAM grafts for mucosal defects. In three of these, hAM was directly sutured to the adjacent mucosa around the defect after excision of the lesion (Lai et al., 1995; Kar et al., 2014; Amemiya et al., 2015). Differently, Khademi et al. (2013) fixed the hAM with sutures to the underlying mucosal membrane. Arai et al. (2012) stabilized the hAM by applying pressure with a dressing of antibiotic ointment gauze, which was removed after 1 week.

Five studies investigated hAM in mandibular vestibuloplasty. After having done Clark’s or Kazandjian’s technique and sutured the mucosal flap to the periosteum, Guler et al. (1997) closed the wound by suturing the hAM graft directly to the adjacent oral mucosa, without further protection in the oral cavity, but with a tight compression dressing over the lower lip. Samandari et al. (2004) performed Clark’s technique and the remaining wound was closed with an hAM graft, sutured to the adjacent oral mucosa. The graft was protected by a splint covered with 3% tetracycline topical gel and stabilized with two pieces of wire, removed after 1 week. Using the same technique, hAM was sutured to the underlying periosteum and stabilized for 1 week with a suction catheter stent (Sharma et al., 2011) or a surgical splint secured with miniature screws (Kothari et al., 2011; Tsuno et al., 2014).

Finally, Rohleder et al. (2013) explored the possibility of using hAM grafts in oronasal fistulae of the hard palate. After freshening the layers of the fistulae, multiple layers of hAM were sutured to the nasal epithelium and subsequently to the oral mucosa of the palate. The whole grafted site was then protected with a palatal plate.

### Graft Storage Format

In our review, hAM was implanted or used as a covering graft in six studies in its cryopreserved format (Velez et al., 2010; Khademi et al., 2013; Rohleder et al., 2013; Kar et al., 2014; Amemiya et al., 2015; Ragazzo et al., 2018) and in 12 studies in its lyophilized or dried format with additional gamma-sterilization in some cases (Guler et al., 1997; Kothiwale et al., 2009; Arai et al., 2012; Shetty et al., 2014; Tsuno et al., 2014; Kiany and Moloudi, 2015; Kumar et al., 2015; Sharma and Yadav, 2015; Jain et al., 2017; Kumar et al., 2017; Kaur and Bathla, 2018; Rehan et al., 2018). A non-usual format of fresh or glycerol-preserved hAM was investigated in four studies (Lai et al., 1995; Samandari et al., 2004; Kothari et al., 2011; Sharma et al., 2011). Non-additional processes (de-epithelization, decellularization, …) were applied to the hAM.

ACM was used only in its dehydrated format in six studies, sometimes de-epithelized and irradiated (Holtzclaw, 2015; Hassan et al., 2017; Cullum and Lucas, 2019; De Angelis et al., 2019; Faraj et al., 2020; Hamada et al., 2020).

### Graft Size and Side

Our analysis of graft sizes found huge variability between the studies. The sizes were different between the tissue banks who commercialized or delivered the membranes. Another factor in this variability was the size of the defects in the oral cavity. The smallest size was 8 by 8 mm to manage alveolar ridge preservation (Cullum and Lucas, 2019); the biggest one was 60 by 35 mm to cover the whole buccal mucosa defect after excision of a lesion (Arai et al., 2012).

Five studies applied the amnion with its mesenchymal side facing the underlying surface (periosteum, mucosa). In surgical wounds after implant surgery, hAM was implanted with its stromal layer in contact with the wound, facilitating its adhesion (Velez et al., 2010). In the management of surgical defects of the oral mucosa, and mainly in mandibular vestibuloplasty, the hAM’s mesenchymal side was placed on the wound or the periosteum (Guler et al., 1997; Samandari et al., 2004; Sharma et al., 2011; Arai et al., 2012).

### Graft Number, Multilayered Use and Folding

When patients have defects in multiple sites, it seems that all the sites were treated at the same time (Cullum and Lucas, 2019) but, mostly, only one site was cured. Four studies reported multilayered hAM or ACM use (Rohleder et al., 2013; Kiany and Moloudi, 2015; Hamada et al., 2020). As previously described, five layers of cryopreserved hAM were used to close oronasal fistulae of the hard palate (Rohleder et al., 2013). In the remainder of the studies, a double layer of membrane was folded around bone substitutes (Holtzclaw, 2015; Kiany and Moloudi, 2015; Hamada et al., 2020).

### Surgical Care and Additional Protective Coverage

Pre- or post-surgical care was somewhat dissimilar between the studies, mainly because of inherent differences in the surgery itself, rather than the hAM application.

Antibiotics (2 g of Amoxicillin, 600 mg of Clindamycin in case of allergy) for 5–10 days and/or 0.12% Chlorhexidine mouth rinses were prescribed before and after hAM/ACM implantation. Sometimes, hydrogen peroxide was recommended because ACM contains bioactive charged proteins that can bind to the chlorhexidine cation and reduce the rate of cellular migration across the membrane (De Angelis et al., 2019; Faraj et al., 2020). Suspension of tooth-brushing was suggested for either 2 weeks (Kiany and Moloudi, 2015; Hamada et al., 2020), 3 weeks (Sharma and Yadav, 2015), or 4 weeks (Rehan et al., 2018) after hAM/ACM implantation. Tap water rinsing or soft toothbrush were recommended in some cases to complete the local hygiene directly after surgery (Kaur and Bathla, 2018; Cullum and Lucas, 2019). Sutures were removed after 1 week in most studies (Kothiwale et al., 2009; Kumar et al., 2015; Kaur and Bathla, 2018); with other studies waiting 10 days (Rehan et al., 2018) or 2 weeks (Kiany and Moloudi, 2015).

When used as covering, pressure was applied to the hAM with a dressing of antibiotic ointment gauze for 1 week (Arai et al., 2012) or a splint covered with 3% tetracycline topical gel protecting the graft for 1 week (Samandari et al., 2004). Nasogastric feeding was initiated for 1 week in two studies (Lai et al., 1995; Kar et al., 2014). Oral feeding was started the day after surgery in two other studies (Arai et al., 2012; Tsuno et al., 2014). When specified, sutures were removed after 1 week (Samandari et al., 2004; Amemiya et al., 2015).

### Graft Re-application and Resorption

Only one application was reported in all these studies, meaning that no re-interventions were needed to apply additional hAM. No information was found about the membrane’s resorption following its ***implantation***. Velez et al. (2010) noticed that cryopreserved hAM induced earlier epithelialization than a standard procedure in wound management around implants, in which patients were their own controls. This speedy healing was shown after 3 days and was significant after 6 days. After 2 weeks, both sides were equivalent in terms of epithelialization. With a de-epithelialized and/or dehydrated ACM, some authors reported epithelialization after approximately 2 weeks (Hassan et al., 2017; Faraj et al., 2020), with mature epithelium visible after 4 weeks (Cullum and Lucas, 2019) and an increase in keratinized tissue width observed at each follow-up visit (7, 15, and 60 days, with the last date being the prosthetic delivery) (De Angelis et al., 2019). With lyophilized hAM, keratinized gingiva was significantly improved between baseline and the 3-month and 6-month post-surgery follow-up visits (Jain et al., 2017).

More information about membrane resorption was found when hAM was used as *a **covering graft*** material. When *fresh or glycerol-preserved hAM* was used, some authors reported persistence of small sections of the membrane after 2 weeks; full resorption after 3 weeks, with the grafted zone being still identifiable at that time (Samandari et al., 2004; Sharma et al., 2011). After 3 months, there were no differences between the grafted sites and the surrounding oral mucosa. Similarly, *dried amnion* was nearly invisible after 1 week (Arai et al., 2012; Tsuno et al., 2014). Guler et al. (1997) could not distinguish *lyophilized hAM* from the surrounding tissues at 10 days post-grafting, with complete resorption being obtained after 2 weeks. After 3 weeks, the grafted zone was covered with oral mucosa. Complete epithelialization was observed in 1 month (Khademi et al., 2013; Amemiya et al., 2015) to 3 months (Kar et al., 2014) after *cryopreserved hAM* grafting; after 3 weeks (with epithelialization continuing at day 10) with the *lyophilized one* (Guler et al., 1997) and after a maximum of 6 weeks associated with a sufficient keratinized gingiva level with the *dried one* (Arai et al., 2012; Tsuno et al., 2014).

Photography was the main evaluation tool used to follow the epithelialization and/or keratinization of the grafted area. Scar contracture information was poorly reported. Some authors evaluated scarring and divided it on a clinical basis (Velez et al., 2010; Arai et al., 2012); others assessed mucosal suppleness (Kar et al., 2014).

X-rays were used to evaluate hAM/ACM GTR/GBR function in the protection of underlying bone substitute (Kiany and Moloudi, 2015; Kumar et al., 2015; Cullum and Lucas, 2019). One study used CT scan to follow bone healing (Ragazzo et al., 2018), another one used histology to assess hAM resorption and epithelialization (Samandari et al., 2004).

Since hAM is widely recognized as relieving pain, some authors evaluated it using a pain scale. Velez et al. (2010) used an analog Likert scale, going from 0 (no pain) to 10 (worst pain imaginable). Other studies used a visual analog scale (VAS) (Kar et al., 2014; Tsuno et al., 2014; Hassan et al., 2017; De Angelis et al., 2019). Some authors rated it in three grades (none/mild, moderate and severe) (Khademi et al., 2013), sometimes also evaluating pain relief (Arai et al., 2012).

## Corollary With Ophthalmological and Wound Healing

### Application Method: Implantation or Covering Options

In the oral surgery field, hAM is more frequently implanted and then covered by the gingiva, instead of being used as covering material left exposed in the buccal cavity. Interestingly, ACM has only been used as an implant. The role of the membrane as a dressing in oral surgery is superimposable to what is reported for ophthalmology and wound healing.

This double-option application is a common point within the ophthalmological field. When used as a “graft” or “inlay technique”, hAM is intended to act as a basement membrane for epithelial regeneration and is placed within the boundaries of the diseased area. When it is used to cover the ocular surface and protect the underlying healing epithelium, it is referred to as a “patch” or “onlay or overlay technique” (Letko et al., 2001; Dua et al., 2004). Erosion or shallow stromal defects in the center of the cornea might be an indication for hAM patch due to optical reasons; for peripheral lesions, an hAM graft might be preferred (Resch et al., 2006).

In our literature review, we noticed that none of implanted hAM/ACM were directly sutured; in two cases the graft’s stability was ensured by sutures (Hassan et al., 2017; De Angelis et al., 2019). From Gulameabasse’s review, implanted ACM was sutured in four studies for stabilization, otherwise it was only applied to the surgical site (Gulameabasse et al., 2020). When hAM was used as a cover, it was sutured in place in 9 of the 11 studies; two studies did not provide enough detail (Guler et al., 1997; Samandari et al., 2004). Suture materials ranged from 4/0 VICRYL^®^ (Sharma et al., 2011; Khademi et al., 2013) to 5/0 polyglactin 910 sutures (Guler et al., 1997) or much thinner sutures such as 7/0 PROLENE^®^, removed 1 week later (Amemiya et al., 2015).

In contrast to hAM application in oral surgery, sutures are always necessary in ophthalmology. The suture material used in conjunction with the hAM is usually 10-0 nylon, 8- to 10-0 VICRYL^®^ or PROLENE^®^. Sutures may be interrupted, running, or mattress type. Mattress sutures are generally placed tangential to the limbus, tacking the membrane to the episclera or superficial sclera. Sutures can be removed at 3 weeks (Dua et al., 2004). In the overlay technique, the patch is secured to the surrounding conjunctiva–episclera with interrupted 8-0 or 9-0 VICRYL^®^ sutures. An additional 10-0 VICRYL^®^ purse-string suture may be placed in the midperipheral cornea (Sippel et al., 2001).

In the ophthalmological field, some improvements had been implemented to avoid hAM suturing and suture removal. The amnion can be kept in place with a tissue adhesive (fibrin glue, gelCORE) or mounted on a plastic structure (Kheirkhah et al., 2008; Kotomin et al., 2015; Shirzaei Sani et al., 2019). Prokera^®^ (Bio-tissue Inc., Miami, FL, United States) is a commercially available medical device that acts as a sutureless biological bandage; it is made of cryopreserved amniotic membrane clipped to a thermoplastic ring set (Kheirkhah et al., 2008). Similarly, in the AmnioClip-plus device, the hAM is clamped in a ring system (Kotomin et al., 2015). All these techniques have several advantages inasmuch as they can be performed under topical anesthesia, the surgery time is shorter and there are no suture-related complications (Kheirkhah et al., 2008). These fixation devices have applications in oral surgery and deserve to be developed, especially when the membrane is used as a coverage material.

On the contrary, no suturing is required for wound healing since the wound allows it to self-adhere. Sometimes, sterile tweezers are used to remove any air bubbles under the hAM to ensure it is in close contact with the wound bed (Valiente et al., 2018). Some authors also blow sterile 42°C air for 5 min to improve adhesiveness (Lo et al., 2010). The amnion is left uncovered and undisturbed (Gajiwala and Gajiwala, 2004) or protected by an additional covering material (see below). Interestingly, fibrin glue (Tissucol Baxter, Vienna, Austria) is used on wounds with a spraying technique to avoid shearing off through manipulations before the hAM is applied (Loeffelbein et al., 2014).

### Graft Size and Side

In oral surgery, graft sizes vary between the studies, depending on the defect size and shape. Small sizes corresponded to lyophilized or dehydrated hAM/ACM; these formats allow for easy cutting. The membranes are often trimmed to match the defect’s shape. Only hAM is applied regardless of the application side when it is implanted (one study) or used as a covering graft material (four studies). We previously reported in an animal model that cryopreserved hAM seemed to induce greater bone formation when the mesenchymal side covered the defect (Fenelon et al., 2020). Although composed of two fetal membranes (amnion and chorion), the side of ACM application is not important (Gulameabasse et al., 2020).

The membrane size used in ophthalmology is obviously much smaller than the one used in oral surgery; however, applying the mesenchymal or epithelial side has some similarities because AM orientation, in these indications, seems to be critical for healing.

In the inlay method, hAM covers the defect after trimming away the excess edges with the epithelial–basement side facing up (Lee and Tseng, 1997). The hAM thereby functions as a basement membrane over which new corneal epithelium can grow (Lee and Tseng, 1997; Sippel et al., 2001). In the overlay technique, depending on the disease severity, it can cover the entire ocular surface (cornea, bulbar, forniceal and palpebral conjunctiva) or just part of it (Dua et al., 2004). In this case, hAM is used primarily to contain the inflammatory reaction while epithelialization is occurring beneath the membrane, which is sutured with its epithelial side against the ocular surface. The mesenchymal side of the hAM traps inflammatory cells and induces apoptosis, thereby reducing inflammation.

The size of the membranes used in wound healing is logically larger since the skin defects are larger, from 2 × 2 cm to 10 × 10 cm (Insausti et al., 2010; Lo et al., 2010; Castellanos et al., 2016; Valiente et al., 2018; Xue et al., 2018). Very few publications reported the membrane’s orientation; when it was specified, it was similar to oral surgery and ophthalmology with the mesenchymal side facing the wound’s surface (Mermet et al., 2007; Insausti et al., 2010). In the study by Valiente et al. (2018), no consideration was given to which side of the hAM was applied when treating diabetic foot ulcer, with positive effects observed when using either side.

### Graft Number, Multilayered Use and Folding

Most of the analyzed studies in the oral surgery field feature single layer hAM application. Because hAM does not have any space maintenance capabilities, some authors suggested using multiple hAM layers and reported its usefulness as a barrier (Rohleder et al., 2013). Two layers of hAM can be folded over bone allograft, delaying its degradation or enhancing its barrier function without any consequences (Holtzclaw, 2015; Kiany and Moloudi, 2015; Hamada et al., 2020). In general, some authors specify that if the membrane folds over itself, it should not be unfolded (Gulameabasse et al., 2020). We recognize that membrane multilayers or folding possibilities are an advantage to consider in oral surgery.

In ophthalmology, except for deep ulcers, one layer of hAM is generally sufficient (Lee and Tseng, 1997). If necessary, two membranes can be used in the same eye: one epithelial side up and the other, epithelial side down. This combines both techniques: inlay graft followed by an overlay patch. In this case, the inner membrane applied to the ocular surface is sutured with the epithelial side up, acting as a graft. The other, usually larger membrane is sutured on top of the first, with the mesenchymal side up. The second membrane acts as a protective bandage for the first membrane and the cells growing on it. The arrangement of layer surfaces is not important except for the uppermost layer which should be placed with the epithelial side up to allow coverage by corneal epithelial cells (Kruse et al., 1999).

Up to 6 layers of hAM can be used to fill a deep defect (Chen et al., 2000; Prabhasawat et al., 2001; Nubile et al., 2011). The hAM is cut into small pieces and placed mesenchymal side down, layer by layer, to fill the ulcer and cover the defect. Usually, the top layer is applied with epithelial side up and secured with 10–0 nylon sutures (Kruse et al., 1999; Hanada et al., 2001). A final hAM is used as a cover with its epithelial side up. The number of applied layers is also very important, because corneal epithelium can grow between the hAM layers (Resch et al., 2006). Along with this multiple individual layer application, hAM may be used as a “fluffed-up” sheet of membrane or as a multilayer sheet (John, 2003). In the latter, the membrane is folded on itself twice which makes it four-layered, or more if needed, much like folding a blanket (“blanket-fold”) and then anchoring it to the cornea. In either case, a second single sheet of hAM is placed over the entire cornea.

As far as we know, multiple layers of hAM are not used for skin defects. In contrast, there are multiple single-layer application times (detailed below).

### Surgical Care and Use of Additional Protective Cover

More than ever, the stability of the hAM/ACM in the oral environment remains to be elucidated. Further investigation is needed to evaluate whether the membrane is robust enough to resist the masticatory and salivary effects for a sufficient time and biodegradable for subsequent repair and maturation of the mucosal tissues. The use of an additional protective cover would be highly recommended, no matter the tissue being healed. It probably also acts on wet environment preservation; humidity being a factor for the success of hAM transplantation (Shimazaki et al., 2000; Santos et al., 2005; Fetterolf and Snyder, 2012).

Kiany and Moloudi (2015) attributed the good stability of hAM, once applied in the oral cavity on the root surface, with a moist environment, with no need for suturing or fixation. Nevertheless, when used as a covering material, hAM may need further protection to delay its degradation or stop it from shifting. Different devices have been evaluated: splints either fixed with bone screws or with sutures, removed after 1 week; palatal plate or pressure dressings, either directly against the hAM or with an extra-oral application; suction catheter stents.

Though all these devices are adequate solutions, splints may prove to be the most useful: they are much more comfortable thus allow the patient to continue oral feeding (instead of a nasogastric tube) and they prevent direct contact between the hAM and the oral environment, thus ensuring good protection. They also offer the possibility of applying a layer of topical gel, which can be an antibiotic or an antiseptic. The same advantages can also be found with palatal plates; 3D printing could have great promise. Additionally, we suggest that, when used as a covering material, hAM needs to be preserved at minima using horizontal mattress sutures with non-absorbable suture. Interestingly, this procedure was applied in one study: after the palatal epithelial-connective graft was configured, the wound was covered with hAM and secured by 3-0 silk non-absorbable mattress sutures (Martelloni et al., 2019).

Like the oral cavity, the eye has a moist environment subject to movement, thereby requiring hAM protection. Depending on the aqueous tear status and the blinking function, a bandage contact lens, hAM as a temporary patch, or temporary tarsorrhaphy was added. The contact lens protects the hAM layers from mechanical and chemical injuries and is removed when the epithelial defect has healed (Chen et al., 2000; Letko et al., 2001).

In wound healing, a large arsenal of protective additional coverage options is available, some of which could be used in the oral surgery field. Some recommendations specify that the graft should be covered with a non-adherent contact layer [e.g., Adaptic (Johnson & Johnson, New Brunswick, NJ, United States) or Mepitel^®^ (Mölnlycke Health Care, Gothenburg, Sweden)] and should not be disturbed, if possible, for at least 1–2 weeks. The secondary dressing environment should be moist, and a moisture management dressing suited to the wound type and treatment is recommended (Fetterolf and Snyder, 2012).

Thus, the following coverings are used: gauge dressing with appropriate splints as indicated (Gajiwala and Gajiwala, 2004); lipidocolloid dressing, UrgoTul^TM^ (Laboratoires Urgo, France) with compression bandages or level II/III compression stockings (Mermet et al., 2007); silicone dressing (Lo et al., 2010); petrolatum gauze and a secondary polyurethane (PU) foam dressing (Alsina-Gibert and Pedregosa-Fauste, 2012); PU foam (Mepilex, Mölnlycke Health Care, Erkrath, Germany) or PU foil (3M^TM^ Tegaderm^TM^ Film, 3M, St. Paul, MN, United States) and consecutive paraffin gauze (Jelonet, Smith & Nephew GmbH, Marl, Germany) (Loeffelbein et al., 2014); petrolatum gauze (Bama-Geve S.L.U, Spain) (Xue et al., 2018); non-adhesive dressing and a crepe bandage (Valiente et al., 2018).

### Graft Re-application and Resorption

#### Re-application

Graft re-application is a common practice in dermatology with great benefits; however, it is not very common in ophthalmology and never done in oral surgery. This wound healing re-application procedure could be adopted in the field of oral surgery, especially when hAM/ACM is used as a covering material. If the ocular surface is heavily inflamed, the membrane disintegrates faster and may have to be reapplied several times (Letko et al., 2001; Kheirkhah et al., 2008). A few authors have reported the re-application of hAM in <15% (Chen et al., 2000; Letko et al., 2001) to 30% of patients, 9–52 weeks after the first one (Resch et al., 2006). During surgery, the hAM parts that remain from the previous membrane transplantation are completely removed (Resch et al., 2006). However, the low reapplication rate could also be due to the fact that fixation with sutures means additional surgical trauma for the patient. In the future, this can be avoided with the sutureless application forms such as AmnioClip-plus.

In the wound healing context, the amnion is replaced when necessary in patients with third degree burns (Gajiwala and Gajiwala, 2004). For the management of resistant vascular ulcers, hAM was re-applied once a week for an average treatment duration of 27 weeks (Pesteil et al., 2007); for venous leg ulcer: three times each 2 weeks if necessary (Lullove, 2017). In diabetic foot ulcers, patients received an average of two applications (range: 1–11) at intervals of 4 week, or 5 weeks or more (Abdo, 2016; Lullove, 2017) or an average of 12 applications (range: 4–40) every 1–2 weeks (Valiente et al., 2018).

#### Resorption

When implanted, the hAM/ACM’s fate in the oral cavity is difficult to analyze because it is not directly visible, and its resorption has not been assessed. A pre-clinical animal study found complete histological resorption of cryopreserved hAM after 4 weeks (Amemiya et al., 2008). De-epithelialized and dehydrated ACM BioXclude^TM^ resorbs in 8–12 weeks, according to the manufacturer, but we were unable to find any proof-of-concept publications. Moreover, Kumar suggests that hAM has excellent acceptability with bone grafts by demonstrating good containment of the material and that it resorbs without the formation of voids and detritus (Kumar et al., 2015). Nevertheless, hAM/ACM effects can be evaluated by direct observation of epithelialization or keratinization in the graft area. Epithelialization begins faster with cryopreserved hAM (from 3 to 6 days, with a mature epithelium at 2 weeks) compared to de-epithelialized and dehydrated ACM (from 2 weeks, with a mature epithelium at 4 weeks). Improvement of keratinized gingiva was only reported for lyophilized hAM and ACM formats (Arai et al., 2012; Tsuno et al., 2014; Jain et al., 2017; De Angelis et al., 2019).

When used as a cover, lyophilized or dried amnion were nearly invisible after 7–10 days; complete resorption was achieved after 2 weeks. When using fresh or glycerol-preserved hAM, full resorption occurred slower, i.e., after 3 weeks. Animals studies showed complete resorption after 2 weeks with a cryopreserved hAM and 5 weeks with a multilayered one (Amemiya et al., 2010; Kesting et al., 2010). Complete epithelialization occurred faster with a lyophilized hAM (3 weeks) than the cryopreserved (4 weeks) or dried form (6 weeks). A good balance between resorption and epithelization is the objective.

Membrane fate is naturally easier to assess in ophthalmology or wound healing area. The formation of an epithelial layer is critical, since failure of proper epithelialization precludes hAM integration in ocular healing (Nubile et al., 2011). It also points to the role of epithelial/stromal interactions in facilitating hAM epithelialization by corneal epithelial cells and corneal stroma-derived cell migration into the transplanted amnion (Resch et al., 2006).

When used as a graft, hAM becomes incorporated into the substratum of the host epithelium (cornea and conjunctiva) and persists for a long time (Dua et al., 2004). Lee reported that resorption occurred between 2 and 14 months follow-up in some patients; for other patients, an afterglow of intact hAM was found after 19 months follow-up (Lee and Tseng, 1997). On the contrary, Letko et al. (2001) reported that the membrane dissolves under the bandage contact lens in 4 weeks after surgery. After applying multilayer hAM, Kruse reported gradual dissolution over a period of 12 months, but stromal thickness remained stable (Kruse et al., 1999), while Hanada reported 15 months (Hanada et al., 2001). Using the overlay, inlay or sandwich surgical techniques, Resch et al. (2006) reported that integrated hAM was found in 18 of 24 corneas up to approximatively 20 months after transplantation.

Used as a patch, the membrane usually falls off, often earlier than desired, or may eventually be removed (Dua et al., 2004). Chen reported that in some cases, hAM dissolved between 10 days and 1 month (Chen et al., 2000); others found 1–2 months after transplantation (Resch et al., 2006).

Mermet et al. (2007) reported that hAM had adhered to the wound bed 1 week after transplantation and the take rate of hAM was 100%. They hypothesized that hAM does not survive in chronic wounds after 2–4 weeks. In one of the review co-author’s experiments, hAM was observed 24 h after its wound application and no signs of its presence were found (unpublished results).

### Common Beneficial Role of hAM/ACM in Oral Mucosa and Skin Wound Healing

The healing of oral mucosal wounds goes through similar stages as that of skin wounds; however it is faster with minimal to no scar formation coupled with a smaller inflammatory response with less neutrophil, macrophage, and T-cell infiltration (Turabelidze et al., 2014). Keratinocyte function is critical for effective wound re-epithelialization, an essential part of the remodeling phase of wound healing. Based on gene expression profiles, proliferation and migration rate of keratinocytes from mucosa is much more rapid than skin keratinocytes (Turabelidze et al., 2014). In wound healing, there is strong evidence about the intimate role of amnion in the stimulation of keratinocyte proliferation and migration because of its effect on the TGFβ signaling pathways (Liarte et al., 2020). These data allow us to envision a very favorable effect of the hAM and this, in a less deleterious environment.

## Graft Storage and Effect on its Properties

There is an on-going debate in the literature about hAM properties that are relevant for oral applications, i.e., mechanical strength, degradation or adhesion properties. This could be explained by how the processing applied to hAM affects its properties. A decellularization treatment is often applied to hAM. It removes the major immunogenic cellular components, membrane-associated antigens, and soluble proteins, thus preventing initiation of a cell-mediated or humoral immune response and subsequent degradation and rejection once implanted into a patient, guaranteeing its antigenicity (Courtman et al., 1994; Wilshaw et al., 2006). It is responsible for a significant decrease in hAM thickness without significantly decreasing its ultimate tensile strength, extensibility, or elasticity (Wilshaw et al., 2006).

Lyophilization or dehydration are often applied to hAM, and they are frequently combined with decellularization and gamma irradiation. One major advantage of lyophilized or (hyper)dried products is that they can be cut easily to the desired size and shape with scissors just before application (Arai et al., 2012; Koob et al., 2016). The second advantage is that the hAM can be preserved for a long time at room temperature without deterioration, simplifying the transport and storage condition and, therefore, decreasing the cost of the product (Ilic et al., 2016; Koob et al., 2016). In addition, the graft is usable as it is, contrary to glycerol-preserved membranes that require thawing and rinsing for approximatively 1 h. Moreover, it returns to a layered structure like that of fresh amnion when it absorbs water, thickens and becomes flaccid, and its transparency increases, suggesting that the membrane may have sufficient strength (Arai et al., 2012). Once hydrated, the tissue matrix is bioactive, can be decorated by matrix metalloproteinases and remodeled by host cells, becoming incorporated into host tissue and eventually replaced with native host tissue (Koob et al., 2016). Lyophilization appears to decrease the thickness and strength of the membrane; however, it improves its adhesion properties compared to fresh and cryopreserved hAM (Niknejad et al., 2011). Gurinsky (2009) reported the ability of processed dehydrated amnion to self-adhere, reducing surgical time and eliminating the need for sutures in the management of gingival recession. Originally designed for ophthalmological use, their current indications have also been extended to wound healing, including diabetic foot, venous leg ulcers and lower third nasal reconstruction (Fetterolf and Snyder, 2012; Koob et al., 2016; Laurent I. et al., 2017; Lullove, 2017; Xue et al., 2018) as well as oral and maxillo-facial surgery as shown in this review.

These modifications of hAM properties are confirmed by recent studies comparing fresh hAM (F-hAM), cryopreserved hAM (C-hAM), lyophilized hAM (L-hAM) and decellularized then lyophilized hAM (D-hAM) (Fenelon et al., 2019; Fenelon et al., 2020). We reported in *in vivo* studies that F-hAM and D-hAM were significantly stronger than C-hAM and L-hAM. We observed that the decellularization process increased the physical and mechanical properties of D-hAM. It made hAM significantly more stretchable than F-hAM, significantly enhancing the tearing strength and significantly decreasing the hAM’s rate of resorption. It also improved *in vitro* and *in vivo* osteogenic potential. It was interesting to note that the cryopreservation process did not affect some of its biomechanical properties (Fenelon et al., 2020). Similarly, repeated freezing procedures impacted cell viability but not histological and ultrastructure analysis (Pogozhykh et al., 2020).

In our literature review, we found that cryopreserved hAM had been tested in six studies, with lyophilized (preferably) and dried formats being tested in 12 clinical studies in total. Cryopreserved hAM was often tested as a covering. In contrast, lyophilized and dried formats were only implanted. Surprisingly, because of the time required to confirm the mother’s status after birth and because of the storage facility, fresh and glycerol-preserved hAM were used as covering graft material in four studies.

The decellularization process, coupled to lyophilization or dehydration, often with additional gamma sterilization, make chorion and ACM a novel and safe option for oral and periodontal surgery.

ACM is only used as an implanted dehydrated membrane in six studies, sometimes de-epithelized and irradiated. Some authors reported that denuded (de-epithelized) hAM promotes better cell proliferation and differentiation, better structural integrity, as well as more uniform cell outgrowth compared to intact hAM (epithelialized) (Koizumi et al., 2000; Koizumi et al., 2007). Hence, it has been the preferred choice for ocular surface reconstruction. In our analysis, de-epithelization of the dehydrated ACM did not contribute to faster epithelization.

In addition to being easy to be manipulate because of its thickness (around 300 μm), ACM also has more growth factors compared to hAM (Koob et al., 2015; McQuilling et al., 2017). Moreover, the decellularization process ensures the safety of chorion and ACM potentially contaminated by maternal cells (Heazlewood et al., 2014; Sardesai et al., 2017). However, although their clinical use has been proven, to our knowledge, their use in Europe is not yet widespread. In this overview, we have chosen not to address the use of chorion since ACM obviously shares some of the same properties (lyophilized or dehydrated format, handling, rate of resorption) and its effectiveness has been described recently (Gulameabasse et al., 2020).

## Conclusion

While the literature has largely described hAM grafting in ophthalmology and wound healing, its application in oral surgery remains a technical challenge. The required surgical procedure has not been sufficiently described or defined in the literature, especially regarding the application side, folding or suturing. When hAM is used as a dressing, there is no consensus about the need for an additional protective coverage, contrary to ophthalmology or wound healing indications. For the eyes, two clear applications exist: “graft or inlay technique” and “patch or onlay/overlay technique.”

We propose a specific nomenclature for hAM/ACM application in the oral cavity beyond the terms “implanted membrane” or “covering graft material” used in this overview:

– “implantation”: the membrane is buried and completely covered by the gum

– “apposition”: the membrane is applied against the site to be treated, not sutured, left exposed in the mouth and stabilized by any means (cross stitches, pressure dressing, palatal plates, etc.)
– “whole covering graft material”: the membrane is applied against the site to be treated, sutured to adjacent mucosa or underlying mucosa, fully left exposed in the mouth and protected by any means (cross stitches, pressure dressing, palatal plates, etc.).

In case of bone exposure, an additional surgery could be defined as “partial covering graft material” where the membrane is applied against the bone, buried under the wound edges, sutured to adjacent mucosa or underlying mucosa, left partially exposed in the mouth and protected by any means (cross stitches, pressure dressing, palatal plates, etc.).

Our overview also highlights that graft storage format does not seem to have any impact on the surgical procedure. Although lyophilized or dehydrated hAM/ACM may facilitate handling and storage without the need for thawing/rinsing, to date, in oral applications, better efficacy in terms of resorption rate, faster re-epithelization or tissue regeneration is not supported by clinical studies. The application size of hAM/ACM is not always rationalized. Neither is the use of a multi-layered graft or re-application, which are a common practice compared to ophthalmology and wound healing, respectively. Together with graft resorption/fate, all this information is essential in terms of the product’s pharmacological.

Pre- and post-surgical care were mainly dependent on the surgery itself rather than the allograft application. The few evaluations tools reported mainly focused on the epithelialization and/or keratinization process, sometimes illustrated by photography, an adequate visual assessment. Imaging was not used enough to exclusively evaluate the membrane’s effectiveness in bone healing; the role of fetal membranes in inducing bone formation cannot be discounted. Histology is poorly described and requires too many invasive acts during the follow-up period.

Given this, we propose some recommendations in Table 3. We believe that in the present overview, the collection of information will assist oral surgeons in hAM/ACM application. Furthermore, as a position paper, we strongly recommend fulfilling the different criteria that we have identified to be as complete as possible in the clinical application of hAM/ACM.

**TABLE 3:** Summary of future recommendations for hAM/ACM application in oral surgery.

**Proposed nomenclature**	**Surgical procedure**	**Graft storage format**	**Side**	**Folding/Multilayers**	**Additional protective cover**	**Re-application**	**Evaluation tools**
**Implantation**	Burry in defect and cover with gum; no membrane suture	Lyophilized or dehydrated and gamma-sterilized ACM advantages	Mesenchymal side when possible	Folding or Multilayer use when necessary	None	Recommended if inflammation persists and/or early re-exposure of the treated site	Epithelialization Keratinization Scar/contracture Imaging if possible to highlight membrane effectiveness Membrane resorption when possible
**Apposition**	Apply to defect; no membrane suture				Stabilized or protected by cross stitches, pressure dressing, palatal plates, etc.		
**Whole covering graft** (membrane left fully exposed in mouth)	Burry under wound edges and suture to adjacent mucosa or underlying mucosa						
*In case of bone exposure*: **Partial covering graft** (membrane left partially exposed in mouth)	Suture to adjacent mucosa or underlying mucosa						

## Author Contributions

SO and FG designed the study and wrote the manuscript. MF assisted the redaction of the 1st section “hAM and ACM Application in Oral Cavity” and revised all the versions of the manuscript. MF, AL, CMe, BC, CMa, SL, NZ, and JC-F contributed to the understanding of hAM and ACM application since they are investigators in an ongoing clinical trial in the field. Moreover, they actively supported the redaction of conclusion and the Table 3. FN, NH, XL, and AS-G assisted the redaction of the 2nd section “Corollary With Ophthalmology and Wound Healing.” NH, HK, FP, and PM assisted the redaction of the 3rd section “Graft Storage and Effects on Its Properties.” All authors contributed to manuscript revision, read, and approved the submitted version.

## Conflict of Interest

The authors declare that the research was conducted in the absence of any commercial or financial relationships that could be construed as a potential conflict of interest.
